# Mercaptopurine versus placebo to prevent recurrence of Crohn's disease after surgical resection (TOPPIC): a multicentre, double-blind, randomised controlled trial

**DOI:** 10.1016/S2468-1253(16)30078-4

**Published:** 2016-08-30

**Authors:** Craig Mowat, Ian Arnott, Aiden Cahill, Malcolm Smith, Tariq Ahmad, Sreedhar Subramanian, Simon Travis, John Morris, John Hamlin, Anjan Dhar, Chuka Nwokolo, Cathryn Edwards, Tom Creed, Stuart Bloom, Mohamed Yousif, Linzi Thomas, Simon Campbell, Stephen J Lewis, Shaji Sebastian, Sandip Sen, Simon Lal, Chris Hawkey, Charles Murray, Fraser Cummings, Jason Goh, James O Lindsay, Naila Arebi, Lindsay Potts, Aileen J McKinley, John M Thomson, John A Todd, Mhairi Collie, Malcolm G Dunlop, Ashley Mowat, Daniel R Gaya, Jack Winter, Graham D Naismith, Holly Ennis, Catriona Keerie, Steff Lewis, Robin J Prescott, Nicholas A Kennedy, Jack Satsangi

**Affiliations:** a Gastrointestinal Unit, Ninewells Hospital, Dundee, UK; b Gastrointestinal Unit, Western General Hospital, Edinburgh, UK; c Colorectal Surgery, Western General Hospital, Edinburgh, UK; d Gastrointestinal Unit, Glasgow Royal Infirmary, Glasgow, UK; e Gastrointestinal Unit, Aberdeen Royal Infirmary, Aberdeen, UK; f Department of Surgery, Aberdeen Royal Infirmary, Aberdeen, UK; g Department of Gastroenterology, Royal Devon and Exeter Hospital NHS Foundation Trust, Exeter, UK; h IBD Pharmacogenetics Unit, University of Exeter, Exeter, UK; i Department of Gastroenterology, Royal Liverpool University Hospital, Liverpool, UK; j Translational Gastroenterology Unit, Nuffield Department of Experimental Medicine, University of Oxford, Oxford, UK; k Department of Gastroenterology, Leeds General Infirmary, Leeds Teaching Hospitals NHS Trust, Leeds, UK; l Department of Gastroenterology, Darlington Memorial Hospital, Darlington, UK; m Department of Gastroenterology, University Hospital Coventry and Warwickshire NHS Trust, Coventry, UK; n Department of Gastroenterology, Torbay Hospital, South Devon Healthcare NHS Foundation Trust, Torbay, Devon, UK; o Department of Gastroenterology, Bristol Royal Infirmary, University Hospitals Bristol NHS Foundation Trust, Bristol, UK; p Department of Gastroenterology, University College London Hospitals NHS Foundation Trust, London, UK; q Department of Gastroenterology, Rotherham NHS Foundation Trust Hospital, Rotherham, UK; r Department of Gastroenterology, Singleton Hospital, Abertawe Bro Morgannwg University Health Board, Swansea, UK; s Department of Gastroenterology, Manchester Royal Infirmary, Central Manchester University Hospitals NHS Foundation Trust, Manchester, UK; t Department of Gastroenterology, Derriford Hospital, Plymouth Hospitals NHS Trust, Plymouth, UK; u Department of Gastroenterology, Hull Royal Infirmary, Hull and East Yorkshire Hospitals NHS Trust, Hull, UK; v Department of Gastroenterology, Royal Stoke University Hospital, University Hospitals of North Midlands NHS Trust, Stoke-on-Trent, UK; w Department of Gastroenterology, Salford Royal NHS Foundation Trust Hospital, Salford, UK; x Department of Gastroenterology, Nottingham University Hospitals NHS Trust, Nottingham, UK; y Department of Gastroenterology, Royal Free London NHS Foundation Trust Hospital, London, UK; z Department of Gastroenterology, Southampton General Hospital, University Hospital Southampton NHS Foundation Trust, Southampton, UK; aa Department of Gastroenterology, Queen Elizabeth Hospital, University Hospitals Birmingham NHS Foundation Trust, Birmingham, UK; ab Department of Gastroenterology, Barts Health NHS Trust, Barts and the London School of Medicine, London, UK; ac Inflammatory Bowel Disease Unit, St Mark's Hospital, North West London Hospitals NHS Trust, London, UK; ad Gastrointestinal Unit, Raigmore Hospital, Inverness, UK; ae Gastrointestinal Unit, Princess Alexandra Hospital, Paisley, UK; af Edinburgh Clinical Trials Unit, University of Edinburgh, Edinburgh, UK; ag Usher Institute, University of Edinburgh, Edinburgh, UK

## Abstract

**Background:**

Up to 60% of patients with Crohn's disease need intestinal resection within the first 10 years of diagnosis, and postoperative recurrence is common. We investigated whether mercaptopurine can prevent or delay postoperative clinical recurrence of Crohn's disease.

**Methods:**

We did a randomised, placebo-controlled, double-blind trial at 29 UK secondary and tertiary hospitals of patients (aged >16 years in Scotland or >18 years in England and Wales) who had a confirmed diagnosis of Crohn's disease and had undergone intestinal resection. Patients were randomly assigned (1:1) by a computer-generated web-based randomisation system to oral daily mercaptopurine at a dose of 1 mg/kg bodyweight rounded to the nearest 25 mg or placebo; patients with low thiopurine methyltransferase activity received half the normal dose. Patients and their carers and physicians were masked to the treatment allocation. Patients were followed up for 3 years. The primary endpoint was clinical recurrence of Crohn's disease (Crohn's Disease Activity Index >150 plus 100-point increase in score) and the need for anti-inflammatory rescue treatment or primary surgical intervention. Primary and safety analyses were by intention to treat. Subgroup analyses by smoking status, previous thiopurines, previous infliximab or methotrexate, previous surgery, duration of disease, or age at diagnosis were also done. This trial is registered with the International Standard Randomised Controlled Trial Register (ISRCTN89489788) and the European Clinical Trials Database (EudraCT number 2006-005800-15).

**Findings:**

Between June 6, 2008, and April 23, 2012, 240 patients with Crohn's disease were randomly assigned: 128 to mercaptopurine and 112 to placebo. All patients received at least one dose of study drug, and no randomly assigned patients were excluded from the analysis. 16 (13%) of patients in the mercaptopurine group versus 26 (23%) patients in the placebo group had a clinical recurrence of Crohn's disease and needed anti-inflammatory rescue treatment or primary surgical intervention (adjusted hazard ratio [HR] 0·54, 95% CI 0·27–1·06; p=0·07; unadjusted HR 0·53, 95% CI 0·28–0·99; p=0·046). In a subgroup analysis, three (10%) of 29 smokers in the mercaptopurine group and 12 (46%) of 26 in the placebo group had a clinical recurrence that needed treatment (HR 0·13, 95% CI 0·04–0·46), compared with 13 (13%) of 99 non-smokers in the mercaptopurine group and 14 (16%) of 86 in the placebo group (0·90, 0·42–1·94; p_interaction_=0·018). The effect of mercaptopurine did not significantly differ from placebo for any of the other planned subgroup analyses (previous thiopurines, previous infliximab or methotrexate, previous surgery, duration of disease, or age at diagnosis). The incidence and types of adverse events were similar in the mercaptopurine and placebo groups. One patient on placebo died of ischaemic heart disease. Adverse events caused discontinuation of treatment in 39 (30%) of 128 patients in the mercaptopurine group versus 41 (37%) of 112 in the placebo group.

**Interpretation:**

Mercaptopurine is effective in preventing postoperative clinical recurrence of Crohn's disease, but only in patients who are smokers. Thus, in smokers, thiopurine treatment seems to be justified in the postoperative period, although smoking cessation should be strongly encouraged given that smoking increases the risk of recurrence.

**Funding:**

Medical Research Council.

## Introduction

Crohn's disease is a chronic, relapsing inflammatory bowel disease. Estimates of the frequency of surgical resection in Crohn's disease vary. Historical data suggest that up to 60% of patients need a major abdominal resection within 10 years of diagnosis.^1^ However, more recent population-based data suggest this figure is as low as 29% at 7 years.^2^ Postoperative recurrence is common within 2 years, in the form of endoscopic signs (72–98% of patients) or clinical symptoms (37–70%), with the proportion of patients needing surgery increasing by 5% per year.^3^, ^4^

**Research in context**

**Evidence before this study**

There remains uncertainty about the efficacy of thiopurines in patients with postoperative Crohn's disease. We searched the Cochrane Central Register of Controlled Trials until May 24, 2016, and PubMed from Jan 1, 1974, to May 24, 2016, with the terms “(azathioprine OR mercaptopurine OR thiopurine) AND Crohn's AND (postoperative OR resection OR hemicolectomy OR ileectomy OR surgical procedures OR surgery) AND trial”, with no language restrictions. We identified two previous systematic reviews with meta-analyses comparing thiopurines with placebo, both published by the Cochrane Collaboration. These reviews differed in their choice of timepoint to assess outcome and in their handling of loss to follow-up. The earlier Cochrane review compared clinical recurrence at a standard time of 12 months across all studies and used the number of patients with a clinical relapse as the outcome measure. Clinical relapse differed significantly between thiopurines and placebo (risk ratio 0·59, 95% CI 0·38–0·92, favouring thiopurines). The more recent Cochrane meta-analysis used the end of study, which varied between 1 year and 2 years, and regarded anyone who did not complete the study as a treatment failure. This study reported a benefit for thiopurines compared with placebo (risk ratio 0·74, 95% CI 0·58–0·94). The Grading of Recommendations Assessment, Development and Evaluation score for the evidence was low. No further published randomised controlled trials were identified that compared thiopurines with placebo since this meta-analysis.

**Added value of this study**

TOPPIC is, to our knowledge, the largest randomised controlled study of thiopurines for postoperative prevention of Crohn's disease, and the largest interventional study of any kind for this indication, with 240 patients enrolled. We found no significant difference between mercaptopurine and placebo for the primary endpoint of clinical recurrence of Crohn's disease (Crohn's Disease Activity Index >150 plus 100-point increase in score) and the need for anti-inflammatory rescue treatment or primary surgical intervention. Smoking was confirmed as the only factor predictive of disease recurrence. A subgroup analysis revealed that mercaptopurine was effective at reducing the incidence of clinical recurrence within 3 years of surgery in smokers, but not in non-smokers (p_interaction_=0·018).

**Implications of all the available evidence**

Combining our data with those included in the previous Cochrane meta-analyses derives a risk ratio of 0·57 (0·38–0·85) in favour of mercaptopurine for the prevention of post-operative Crohn's disease (appendix p 11). We confirm that smoking affects the clinical course of Crohn's disease, as well as response to treatment, whereas no differences were reported by age, sex, or a history of previous surgery. Smoking cessation should be a priority in patients with Crohn's disease after surgery.

Strategies to prevent or delay postoperative recurrence of Crohn's disease are of major clinical importance. However, there is a paucity of evidence to support any particular drug treatment strategy.^5^, ^6^ Azathioprine and mercaptopurine have an established role in inducing remission, and in the maintenance of medically induced remission, in patients with Crohn's disease. These drugs are recommended in treatment algorithms for patients at high risk of postoperative relapse,^4^ but the evidence to support this, and the evidence that clinical parameters can predict patients at high risk of relapse, is weak. A meta-analysis^7^ showed that efficacy data for thiopurines in this setting were inconclusive and, aside from smoking, there were no consistent predictors of postoperative relapse. A Cochrane review^8^ also concluded that the evidence supporting thiopurines for the reduction of endoscopic and clinical recurrence was insufficient because of the small numbers of patients included and flawed study designs. The value of thiopurine metabolites in postoperative Crohn's disease is unknown. The role of biological treatments in postoperative Crohn's disease has received substantial attention. After smaller randomised studies of infliximab,^9^, ^10^ findings from the POCER study^11^ showed that targeted escalation of immune-modulatory treatment (ie, thiopurines followed by adalimumab) in patients with early endoscopic evidence of recurrence might delay subsequent endoscopic, although not clinical, recurrence.

We therefore aimed to establish whether mercaptopurine, compared with placebo, can prevent or delay postoperative clinical recurrence of Crohn's disease that needs anti-inflammatory rescue treatment or surgery.

## Methods

### Study design and participants

TOPPIC was a randomised, placebo-controlled, double-blind study done at 29 secondary and tertiary UK hospitals. The study was approved by the Scotland “A” Research Ethics Committee.

Patients aged at least 16 years (Scotland) or 18 years (England and Wales) who had a diagnosis of Crohn's disease^12^ and an ileocolic or small bowel resection within the preceding 3 months were eligible for inclusion. Key exclusion criteria were residual active Crohn's disease present after surgery, known intolerance or hypersensitivity to thiopurines, known need for further surgery, strictureplasty alone, formation of a stoma, active or untreated malignancy, absent thiopurine methyltransferase activity, substantial abnormalities of liver function tests or full blood count, and pregnancy. The appendix (p 4) lists all inclusion and exclusion criteria. Before randomisation, postoperative infections were treated and existing treatments for Crohn's disease stopped. The protocol was amended on Sept 28, 2010, to include patients successfully treated for a malignancy and in remission for at least 5 years and to exclude those receiving treatment for active Crohn's disease at random allocation. All patients provided written informed consent before enrolment.

### Randomisation and masking

Patients were randomly assigned (1:1) to mercaptopurine or identical matched placebo using a computer-generated web-based randomisation system managed by the Edinburgh Clinical Trials Unit (University of Edinburgh, Edinburgh, UK) and stratified according to smoking status at baseline and recruiting site (block sizes of two or four). Patients' details were entered into the randomisation system before random allocation and were concealed at randomisation. Patients and their carers and physicians were masked to the treatment allocation. Blood monitoring results were reviewed by an independent central clinician masked to treatment allocation and to mean corpuscular volume results. The appendix (p 5) details the dose reduction algorithm. To protect masking, investigators were informed that sham dose reductions were planned for patients on placebo. However, on the advice of the data monitoring committee, sham dose reductions did not occur; the investigators were not informed of this.

### Procedures

Patients received once daily oral mercaptopurine, at a dose of 1 mg/kg bodyweight rounded to the nearest 25 mg, or identical matched placebo. Patients with low thiopurine methyltransferase activity were prescribed half the normal dose.

Baseline assessments included Crohn's Disease Activity Index (CDAI); patient-reported outcome measures, including the Inflammatory Bowel Disease Questionnaire (IBDQ); a physical examination; and a blood sample for 6-thioguanine nucleotide concentrations (6-thioguanine and 6-methylmercaptopurine; appendix p 6). We also took additional blood samples for genetic and serological analysis and will report those results separately. Treatment was planned for 3 years, with dose adjusted for changes in bodyweight. The appendix (p 6) describes which procedures were done at which timepoints. Blood monitoring was done weekly for the first 6 weeks and thereafter at 6-weekly intervals. Patients with abnormal results had a dose reduction, temporary cessation, or cessation as per a study algorithm (appendix p 5). Patients with persistent nausea or persistent influenza-like symptoms also received a dose reduction, according to the protocol. If abnormal parameters improved after a temporary cessation, treatment was recommenced at a lower dose. At each study visit, the following data were collected: CDAI, physical examination, concomitant medications, and patient-reported outcomes, including the IBDQ (appendix p 6). Samples for assay of faecal calprotectin, 6-thioguanine, and 6-methylmercaptopurine were collected at randomisation and weeks 13, 49, 103, and 157. Faecal samples were stored on site at −80°C and then shipped on dry ice to a central laboratory (Gastrointestinal Laboratory, Western General Hospital, Edinburgh, UK) for analysis with the CALPRO Calprotectin ELISA test (Calpro AS, Lysaker, Norway). Samples were stored in a freezer until the patient exited the study, and all samples for an individual were then tested at the same time. The Edinburgh laboratory has a coefficient of variation of 10% for faecal calprotectin (based on assessments of the entire sample processing pipeline). 6-thioguanine nucleotide and 6-methylmercaptopurine were analysed by the Viapath Purine Research Laboratory (St Thomas' Hospital, London, UK) using a method adapted from Dervieux and colleagues.^13^ Briefly, thioguanine and methylated mercaptopurine nucleotides in whole blood were hydrolysed to the base by boiling in 15% perchloric acid and detected by ultraviolet absorption after separation on a Waters Ultra-Performance Liquid Chromatography system (Waters Limited, Elstree, Hertfordshire, UK). Colonoscopy was done at 49 and 157 weeks after randomisation.

### Outcomes

The primary outcome was clinical recurrence, defined as a CDAI score of over 150 and a 100-point increase from baseline, and the need for anti-inflammatory rescue treatment or primary surgical intervention. Secondary outcomes were clinical recurrence, defined as reaching either of the individual components of the primary outcome (ie, either a CDAI score of >150 and a 100-point increase from baseline, or the need for anti-inflammatory rescue treatment or primary surgical intervention); endoscopic recurrence, defined as a Rutgeerts score of at least i2; Crohn's Disease Endoscopic Index of Severity (CDEIS) score;^14^, ^15^ and quality of life, measured by changes in IBDQ scores. Adverse events were assessed by investigators at the participating sites using criteria defined in the trial protocol.

### Statistical analysis

A sample size of 234 patients was needed to give 80% power to detect a reduction in the frequency of recurrence from 50% in the placebo group to 30% in the treatment group by 3 years at the 5% level of significance.

Analyses were by intention to treat. For the primary analysis, we used a Cox proportional hazards model with terms for treatment and the variables on which the randomisation was stratified (smoking status and recruitment site), adjusted for baseline values of previous treatment with mercaptopurine and previous treatment with azathioprine. We present adjusted and unadjusted Cox proportional hazard ratios (HRs) for the comparison of mercaptopurine versus placebo (reference), with an HR of less than one suggesting a treatment effect in favour of mercaptopurine. For both primary and secondary outcomes, the adjusted analysis was judged to be the primary analysis. We did predefined subgroup analyses of the primary and secondary outcomes to assess treatment effect in terms of previous medical treatment, previous surgery, smoking status, duration of disease, and age at diagnosis. The interaction between subgroup and treatment was included in the Cox regression model to establish whether the treatment effect differed by subgroup.

We compared endoscopic recurrence between treatment groups using a χ^2^ test. CDEIS results at week 157 were compared between treatment groups using a two-sided *t* test. The same subgroups analysed for the primary and secondary outcomes were also analysed with respect to endoscopic recurrence and CDEIS scores. We produced receiver operating characteristic (ROC) curves to calculate the diagnostic accuracy of faecal calprotectin to predict endoscopic recurrence and remission. The optimum cutoff point was calculated by maximising Youden's *J* statistic. We incorporated faecal calprotectin and 6-thioguanine separately into a Cox proportional hazards model as time-varying covariates. Quality of life, as measured by the IBDQ, was analysed using a change from baseline repeated measures ANCOVA to assess the effect of treatment over time for the overall mean IBDQ score and also the overall total IBDQ score. Quality of life as measured by the EQ-5D system was summarised by treatment group across study visits.

We excluded missing data from any formal statistical analyses, with the exception of a secondary sensitivity analysis to the complete case analysis of IBDQ data, for which we used several imputation techniques, as described in the statistical analysis plan. A data monitoring committee oversaw the trial. Data were analysed in SAS version 9.4.

In a post-hoc analysis, we used the absolute risk reduction experienced by patients on mercaptopurine versus placebo to calculate the number needed to treat (NNT), for benefit and harm, for smokers and non-smokers.

This trial is registered with the International Standard Randomised Controlled Trial Register (ISRCTN89489788) and the European Clinical Trials Database (EudraCT number 2006-005800-15).

### Role of the funding source

The funder of the study had no role in study design, data collection, data analysis, data interpretation, or writing of the report. The corresponding author had full access to all the data in the study and had final responsibility for the decision to submit for publication.

## Results

**Figure 1:**
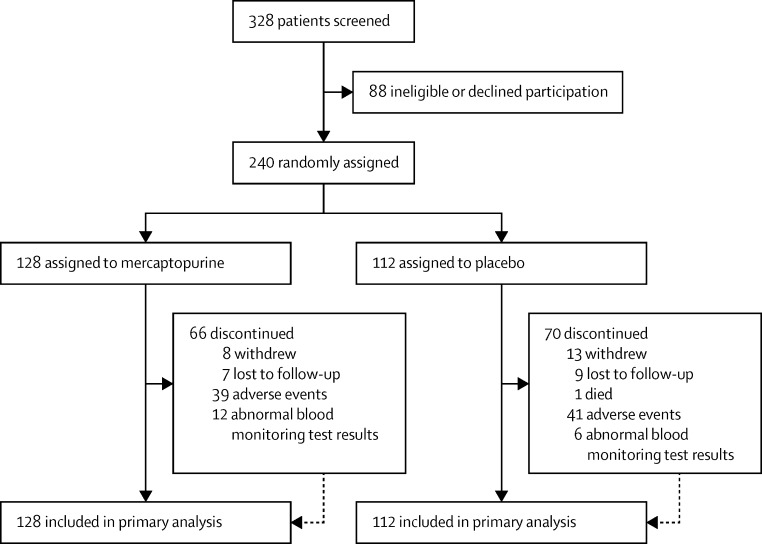
TOPPIC trial profile

Between June 6, 2008, and April 23, 2012, 328 patients were screened at 29 centres (appendix p 7), 240 of whom met eligibility criteria, consented to inclusion, and were enrolled and randomly assigned: 128 to mercaptopurine and 112 to placebo (figure 1). There was low patient recruitment (only one or two patients) at several centres, which resulted in only one treatment being assigned at these centres, which created the imbalance in recruitment numbers between treatment groups. All patients received at least one dose of study drug. 146 (61%) were women and 55 (23%) were smokers (table 1). Baseline characteristics were similar between study groups (table 1). 104 (43%) of 240 patients received study drug at the initial dose for the entire 3-year treatment period. The mean treatment period was 22·6 months (SD 13·7): 23·4 months (14·0) in the mercaptopurine group versus 21·8 months (13·4) in the placebo group. 50 (39%) of 128 patients in the mercaptopurine group and 18 (16%) of 112 in the placebo group had a dose reduction in accordance with the trial protocol. Study drug was discontinued in 66 (52%) of 128 patients in the mercaptopurine groups versus 70 (63%) of 112 in the placebo group for the following reasons: adverse events in 80 patients (59%; 39 in the mercaptopurine group and 41 in the placebo group), abnormal blood test results in 18 (13%; 12 and six), early withdrawal in 21 (15%; eight and 13), loss to follow-up in 16 (12%; seven and nine), and death in one (1%; in the placebo group). The appendix (p 8) summarises data completeness for the study visits. Median follow-up was 36·0 months (IQR 27·5–36·0) in the mercaptopurine group and 36·0 months (19·5–36·0) in the placebo group.

**Table 1:** Demographics and baseline characteristics before randomisation

		**Mercaptopurine (n=128)**	**Placebo (n=112)**
**Sex**			
Female		79 (62%)	67 (60%)
Male		49 (38%)	45 (40%)
**Age (years)**			
Mean (SD)		39·2 (12·8)	38·2 (13·4)
Median (IQR)		38 (28–50)	36 (28–48)
Range		17–67	17–75
**Age at diagnosis**			
≤40 years		103 (80%)	87 (78%)
>40 years		25 (20%)	23 (21%)
Unknown		0	2 (2%)
**Present smoker**			
Yes*		29 (23%)	26 (23%)
No		99 (77%)	86 (77%)
**Duration of Crohn's disease from diagnosis**			
≤1 year		37 (29%)	41 (37%)
>1 year		91 (71%)	69 (62%)
Unknown		0	2 (2%)
**Duration of Crohn's disease from diagnosis (years)**†			
Mean (SD)		7·7 (9·7)	7·6 (9·5)
Median (IQR)		3 (0–11)	4 (0–11)
Range		0–39	0–47
**Crohn's disease location**‡			
Ileal		54 (42%)	39 (35%)
Colonic		4 (3%)	2 (2%)
Ileocolonic		70 (55%)	70 (63%)
**Previous treatments**			
Mercaptopurine‡			
	Yes	14 (11%)	5 (4%)
	No	114 (89%)	106 (95%)
Azathioprine‡			
	Yes	80 (63%)	47 (42%)
	No	48 (38%)	64 (57%)
Either thiopurine‡			
	Yes	81 (63%)	50 (45%)
	No	47 (37%)	61 (54%)
Infliximab§			
	Yes	21 (16%)	15 (13%)
	No	104 (81%)	96 (86%)
Methotrexate‡			
	Yes	8 (6%)	7 (6%)
	No	120 (94%)	104 (93%)
Other corticosteroids‡			
	Yes	97 (76%)	79 (71%)
	No	31 (24%)	32 (29%)
Any immunosuppressants‡			
	Yes	112 (88%)	86 (77%)
	No	16 (13%)	25 (22%)
Previous surgery‡			
	Yes	46 (36%)	28 (25%)
	No	82 (64%)	83 (74%)

Data are number (%), unless otherwise specified. Some percentages do not add up to 100 because of rounding.

* Smoked >1 cigarette per day at study entry.

† Data missing for two patients in the placebo group.

‡ Data missing for one patient in the placebo group.

§ Data missing for three patients in the mercaptopurine group and one in the placebo group.

**Figure 2:**
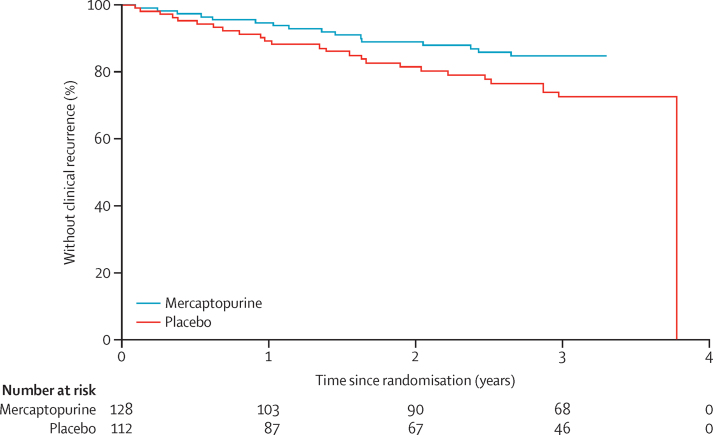
Kaplan-Meier plot for the primary outcome of time to clinical recurrence of postoperative Crohn's disease

**Figure 3:**
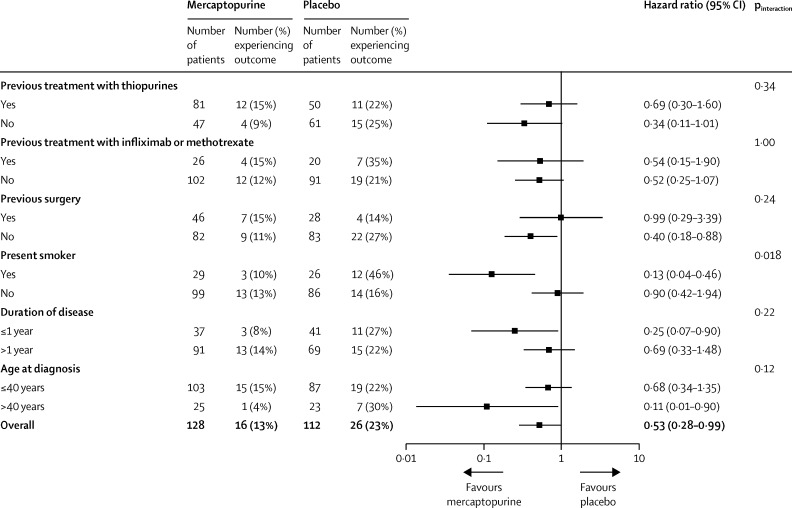
Unadjusted subgroup analyses of the primary outcome of clinical recurrence of postoperative Crohn's disease

The primary endpoint of clinical recurrence of Crohn's disease and the need for anti-inflammatory rescue treatment or primary surgical intervention occurred in 42 (18%) of 240 patients: 16 (13%) of 128 in the mercaptopurine group versus 26 (23%) of 112 in the placebo group (adjusted HR 0·54, 95% CI 0·27–1·06; p=0·07; unadjusted HR 0·53, 95% CI 0·28–0·99; p=0·046; figure 2). All 42 patients met the CDAI criteria for recurrence and had rescue treatment, five (12%) of whom subsequently went on to have surgery. In predefined subgroup analyses, 15 (27%) of 55 smokers had a clinical recurrence, three (10%) of 29 in the mercaptopurine group and 12 (46%) of 26 in the placebo group (HR 0·13, 95% CI 0·04–0·46), compared with 27 (15%) of 185 non-smokers, 13 (13%) of 99 in the mercaptopurine group and 14 (16%) of 86 in the placebo group (0·90, 0·42–1·94; p_interaction_=0·018; figure 3). In a post-hoc analysis, the NNT for benefit was calculated as three for smokers (95% CI 1·7–7·3) and 31 for non-smokers (95% CI NNT_benefit_ 7·5 to ∞ to NNT_harm_ 14·1) across the entire follow-up period. Previous exposure to treatment, previous surgery, thiopurine status, duration of disease, and age at diagnosis had no effect on the response to study drug (figure 3).

**Figure 4:**
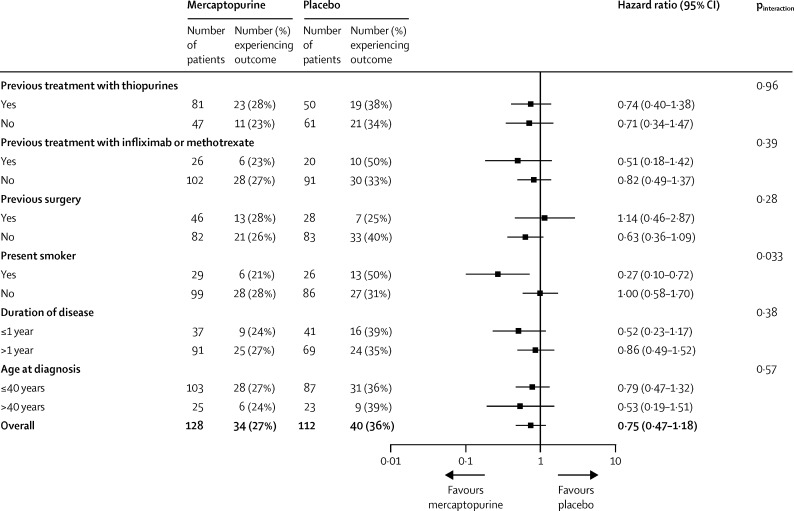
Unadjusted subgroup analyses of the secondary outcome of clinical recurrence

34 (27%) of 128 patients in the mercaptopurine group versus 40 (36%) of 112 in the placebo group experienced the secondary endpoint of clinical recurrence, defined as a CDAI rise or need for rescue treatment or surgery (adjusted HR 0·74, 95% CI 0·44–1·23; p=0·24; unadjusted 0·75, 0·47–1·18; p=0·21). In subgroup analyses, mercaptopurine reduced recurrence in smokers only (p_interaction_=0·033; figure 4).

Of the 208 patients who remained in the study 49 weeks after randomisation, 172 attended for colonoscopy (95 in the mercaptopurine group and 77 in the placebo group), and a Rutgeerts score was available for 168 (91 in the mercaptopurine group and 77 in the placebo group). Of these, 121 (72%; 58 in the mercaptopurine group and 63 in the placebo group) had some form of endoscopic recurrence (score >i0). Of the 161 patients who remained in the study at week 157 after randomisation, 128 (69 in the mercaptopurine group and 59 in the placebo group) underwent a colonoscopy, and a Rutgeerts score was available for 124 (67 in the mercaptopurine group and 57 in the placebo group). Of these, 95 (77%; 47 in the mercaptopurine group and 48 in the placebo group) had some form of endoscopic recurrence (score >i0). 29 (43%) of 67 patients in the mercaptopurine group and 28 (49%) of 57 in the placebo group had endoscopic recurrence with a Rutgeerts score of i2 or greater (p=0·38). We noted a similar pattern at week 49, although this pattern was not formally analysed. Week 157 CDEIS scores did not differ significantly between groups (data not shown). Similarly, none of the CDEIS subgroup analyses showed a significant interaction with treatment (data not shown).

Of the 168 patients who had a Rutgeerts score calculated at week 49, faecal calprotectin concentrations were available in 126 patients (71 in the mercaptopurine group and 55 in the placebo group). Of the 124 patients who had a Rutgeerts score calculated at week 157, faecal calprotectin concentrations were available in 88 patients (46 in the mercaptopurine group and 42 in the placebo group; appendix p 12). These data were combined to generate ROC curves to examine test accuracy at predicting endoscopic recurrence and remission. In both scenarios, the faecal calprotectin measurement proved to be an unreliable test, with an area under the curve of 0·70 (95% CI 0·63–0·77) for recurrence and 0·66 (0·58–0·75) for remission. Selecting a faecal calprotectin concentration of 50 μg/g (commonly proposed as an appropriate cutoff concentration to detect inflammation) to predict endoscopic recurrence produced a sensitivity of 84·4% (95% CI 77·0–91·9), specificity of 44·4% (35·6–53·1), positive predictive value (PPV) of 52·4% (44·3–60·5), and negative predictive value (NPV) of 79·7% (70·2–89·2). Increasing the cutoff concentration to 100 μg/g produced a sensitivity of 72·2% (95% CI 63·0–81·5), specificity of 62·1% (53·6–70·6), PPV of 58·0% (48·9–67·2), and NPV of 75·5% (67·1–83·8). The NPV for the prediction of endoscopic remission with a faecal calprotectin concentration of 50 μg/g was 81·4% (95% CI 75·0–87·7) and with a concentration of 100 μg/g it was 83·9% (77·1–90·7; appendix p 9). Analysis of faecal calprotectin as a time-varying covariate suggested that, for every 100-unit increase in faecal calprotectin, the HR for the primary endpoint (data available for 31 [74%] of 42 patients who reached the primary endpoint) increased by 18% (HR 1·18, 95% CI 1·08–1·28; p=0·0002).

102 (92%) of 111 patients had 6-thioguanine nucleotide concentrations measured at week 49, and 64 (72%) of 89 who remained on mercaptopurine had concentrations measured at week 157. 6-thioguanine nucleotide concentrations were grouped according to the target therapeutic range (235–450 pmol per 8 × 10^8^ red blood cells). At week 49, 61 (60%) of 102 patients had subtherapeutic concentrations, versus 40 (63%) of 64 at week 157 (appendix p 13). In the corresponding time-varying covariate analysis of 6-thioguanine nucleotide concentrations in patients receiving mercaptopurine, the association with the primary outcome was not significant (HR 0·80, 95% CI 0·57–1·13; p=0·21).

IBDQ data were available for 203 (85%) of 240 randomly assigned patients at week 49 (109 in the mercaptopurine group and 94 in the placebo group) and 155 (65%) at week 157 (86 in the mercaptopurine group and 69 in the placebo group). Overall mean or total IBDQ scores did not seem to differ significantly between groups (data not shown).

The incidence and types of adverse events were similar in the mercaptopurine and placebo groups (table 2 and appendix p 10). Adverse events caused discontinuation of treatment in 80 (33%) of 240 patients: 39 (30%) of 128 in the mercaptopurine group versus 41 (37%) of 112 in the placebo group. Of the 1747 reported adverse events, four (<1%) were malignancies (three in the mercaptopurine group [two cases of lentigo maligna in the same individual and one case of basal cell carcinoma] and one in the placebo group [breast cancer]), and one patient on placebo died of ischaemic heart disease. 171 (18%) of 947 events in the mercaptopurine group and 184 (23%) of 798 in the placebo group were infections. Mercaptopurine was temporarily stopped in 32 patients because of abnormal blood monitoring results or other side-effects. Placebo was temporarily discontinued in 35 patients for similar reasons.

**Table 2:** Adverse events

		**Mercaptopurine (n=128)**				**Placebo (n=112)**			
		Mild	Moderate	Severe	Total	Mild	Moderate	Severe	Total
Cancers		0	2 (2%)	0	2 (2%)	0	0	1 (1%)	1 (1%)
Abnormal liver function test		0	4 (3%)	0	4 (3%)	0	5 (4%)	0	5 (4%)
Gastrointestinal symptoms									
	Abdominal pain	15 (12%)	32 (25%)	19 (15%)	66 (52%)	10 (9%)	42 (38%)	15 (13%)	67 (60%)
	Constipation or diarrhoea	12 (9%)	19 (15%)	6 (5%)	37 (29%)	7 (6%)	23 (21%)	7 (6%)	37 (33%)
	Nausea or vomiting	13 (10%)	24 (19%)	8 (6%)	45 (35%)	12 (11%)	16 (14%)	2 (2%)	30 (27%)
	Other	14 (11%)	16 (13%)	4 (3%)	34 (27%)	12 (11%)	16 (14%)	0	28 (25%)
Headache		9 (7%)	17 (13%)	0	26 (20%)	6 (5%)	11 (10%)	3 (3%)	20 (18%)
Infections		31 (24%)	45 (35%)	5 (4%)	81 (63%)	24 (21%)	38 (34%)	6 (5%)	68 (61%)
Joint pain or arthralgia		13 (10%)	23 (18%)	4 (3%)	40 (31%)	8 (7%)	26 (23%)	2 (2%)	36 (32%)
Other		22 (17%)	55 (43%)	8 (6%)	85 (66%)	18 (16%)	35 (31%)	9 (8%)	62 (55%)
Pain		7 (5%)	8 (6%)	3 (2%)	18 (14%)	4 (4%)	10 (9%)	3 (3%)	17 (15%)
Pancreatitis		0	1 (1%)	0	1 (1%)	0	0	1 (1%)	1 (1%)
Rash		14 (11%)	9 (7%)	1 (1%)	24 (19%)	12 (11%)	2 (2%)	0	14 (13%)
Worsening of Crohn's disease		6 (5%)	13 (10%)	5 (4%)	24 (19%)	3 (3%)	20 (18%)	6 (5%)	29 (26%)

Data are number of patients with one or more adverse event in that category. Patients who had more than one adverse effect in the same category but different severity are counted in the most severe category.

14 pregnancies were reported during the course of the trial, with 12 healthy outcomes (ie, successful birth and healthy infant). One spontaneous abortion occurred at about 21 weeks gestation in the mercaptopurine group and one congenital anomaly (heart murmur, septal defect, and hydrocephalus) occurred in a child born to a patient in the placebo group.

In a post-hoc analysis, complete endoscopic remission (Rutgeerts score i0) was maintained in proportionally more patients on mercaptopurine than placebo at both week 49 and week 157 (appendix pp 14–15). In a subgroup analysis, mercaptopurine was more effective at preventing endoscopic recurrence in patients with previous thiopurine exposure (odds ratio 0·25, 95% CI 0·09–0·70) than in thiopurine-naive patients (3·00, 1·00–9·04; p=0·001) Endoscopic recurrence, defined as Rutgeerts score greater than i0 (ie, anything other than complete remission), was present in 58 (64%) of 91 patients in the mercaptopurine group versus 62 (82%) of 76 in the placebo group at week 49 (p=0·01), and in 47 (70%) of 67 in the mercaptopurine group versus 48 (84%) of 57 in the placebo group at week 157 (p=0·07).

## Discussion

To our knowledge, this is the largest randomised, double-blind study to assess the efficacy of mercaptopurine in the prevention of postoperative Crohn's disease. The primary outcome of clinical recurrence of Crohn's disease (Crohn's Disease Activity Index >150 plus 100-point increase in score) and the need for anti-inflammatory rescue treatment or primary surgical intervention, occurred in 13% of patients in the mercaptopurine group versus 23% in the placebo group (adjusted p=0·07); however, clinical recurrence was significantly more common in smokers, in whom mercaptopurine proved beneficial, with an NNT of three. The secondary outcome of endoscopic recurrence was recorded in a third of patients in each group, with no significant difference between groups; however, in a post-hoc analysis, mercaptopurine was significantly more effective than placebo at maintaining complete endoscopic remission. Although there was no significant difference in the prespecified primary clinical efficacy endpoint, the subgroup analyses provide relevant insights in terms of clinical prediction of response and outcome, and in terms of the challenges in assessing outcome by endoscopic or clinical criteria.

Findings from this study confirm that smoking affects the clinical course of Crohn's disease, as well as response to treatment. Of the factors assessed, the primary endpoint was only significantly different between smokers and non-smokers, whereas no differences were reported by age, sex, a history of previous surgery, duration of disease, previous treatments, or thiopurine status. The data highlight the importance of smoking cessation in disease management and support findings from previous studies^16^, ^17^, ^18^ that showed that surgical recurrence increases with the number of cigarettes smoked each day, and that smoking cessation reduces clinical and surgical recurrence. In the present study, treatment with mercaptopurine to delay or prevent postoperative recurrence was particularly effective in people who continue to smoke; thus, in smokers, thiopurine treatment seems to be justified in the early postoperative period. In non-smokers, the data do not provide a sufficiently strong rationale for immediate initiation of treatment in the postoperative period. A considered approach involving colonoscopy in the first 6–12 months is likely to be beneficial in this group.

This study is one of the largest to report on endoscopic recurrence of Crohn's disease, and is important for several reasons. First, the incidence of any endoscopic recurrence was 77% at 3 years, which is similar to the 85% reported previously.^14^ Second, over a 3-year period, there was a poor association between endoscopic and clinical recurrence. There are several possible explanations for this finding, and there is no consensus as to whether to prioritise clinical outcomes over endoscopic outcomes. Third, mercaptopurine seems to maintain complete endoscopic remission (i0), whereas using a cutoff score of at least i2 to define endoscopic recurrence revealed no difference between treatment and placebo groups. Fourth, our study is, to our knowledge, the largest comprehensive assessment of faecal calprotectin in postoperative Crohn's disease. Using a cutoff of 50 μg/g, the NPV for recurrence was 79·7%, which decreased to 75·5% by increasing the cutoff to 100 μg/g. The corresponding NPVs for the prediction of endoscopic remission were 81·4% and 83·9%, respectively. If mucosal integrity is the goal, these values might not provide the confidence to abandon endoscopic assessment. The performance of faecal calprotectin was poorer than reported in the POCER study (NPV of 94%).^11^ Reported differences between studies are probably due to differences in study methods, since in the POCER study, an endoscopic score of i2 was imputed for all missing values; no imputations were made in our study.

Several important factors should be considered when interpreting these findings. Based on existing data, power calculations estimated a 20% difference between mercaptopurine and placebo groups. Clinical recurrence rates were 23·2% in the placebo group and 12·5% in the treatment group: a difference of 10·7%. These rates are lower than those in studies by Hanauer and colleagues^19^ (50% treatment and 77% placebo) and Ardizzone and colleagues^20^ (17% treatment and 28% control), on which the power calculations were based. This marked difference between recurrence rates is probably a result of differing primary outcome definitions; clinical scoring systems advocated to identify disease relapse in clinical trials such as CDAI have flaws, especially in the postoperative setting.^21^ We used an outcome that was based on a disease activity score (CDAI >150 and a 100-point increase from baseline) and the need for medical treatment. In view of the difficulties of using the CDAI postoperatively, we judged this definition of clinical recurrence to be robust. The Rutgeerts score attempts to make the assessment of endoscopic recurrence an objective exercise, but has never been prospectively validated.^14^ We selected a score of at least i2 as a secondary endpoint, in line with previous studies, including the study by Hanauer and colleagues.^19^ However, the limitations of this approach are well recognised; there is little difference between i1 (≤5 aphthous ulcers) and i2 (>5 aphthous ulcers or larger lesions confined to the anastomosis), and inter-observer variation is an issue. In a substudy^22^ of the TOPPIC trial, inter-observer agreement on 43 endoscopic images was measured by five investigators; complete agreement occurred in only 79% of cases. Although centralised reading could overcome some of the inter-observer variation in endoscopic scoring in future studies, an alternative approach might be to regard complete mucosal healing as the preferred therapeutic target in Crohn's disease, and to identify maintenance of endoscopic remission (i0) as the target in postoperative Crohn's disease. A review of the scoring of endoscopic recurrence is warranted.

Optimum dosing in all patients was also difficult to achieve in the context of a double-blind study and a protocol-led dose adjustment strategy. Of the 240 patients, 104 (43%) received treatment at the initial dose for the duration of the study. Data available at the end of the study show that about 60% of patients randomly assigned to mercaptopurine were on subtherapeutic doses, and a stronger treatment effect might have been noted had 6-thioguanine nucleotide results been available to optimise the dose during the study. The rates of discontinuation of treatment and withdrawal or loss to follow-up in this study are similar to those in previous work. For example, Ardizzone and colleagues^20^ reported treatment discontinuations owing to adverse events in 15 (22%) of 69 patients in the azathioprine group versus six (9%) of 69 in the mesalazine group, although data on treatment discontinuation within other trials are not clearly documented. Analysis of these data was on an intention-to-treat basis and therefore the effect of the drug taken at full dose in an individual patient is likely to have been underestimated. In clinical practice, many patients taking thiopurines might be inadvertently under-dosed initially, but are identified on the basis of mean corpuscular volumes or, more recently, available metabolite testing.^23^

Adverse events were noted frequently in both groups but were generally mild. Rates of pancreatitis and malignancy were lower than expected. Unusually for a clinical trial, we did not remove patients who became pregnant during the trial, in keeping with accepted clinical practice. We noted 14 pregnancies, with 12 healthy outcomes. No fetal malformations occurred in the mercaptopurine-treated group. Masked safety monitoring contributed to the validity of the results.

The strengths of this study include the double-blind design, the comparison of symptom scores with endoscopic findings, the assessment of faecal calprotectin in a large number of patients in the postoperative setting, and a demonstration of the potential usefulness of 6-thioguanine nucleotide concentrations in patient management. The study also included patients from 29 UK centres, both secondary and tertiary hospitals, which makes it generalisable beyond specialist centres. Limitations include the absence of therapeutic drug monitoring with dose adjustment, missing colonoscopy data in 20% of eligible patients, and the absence of centralised endoscopy reading. Furthermore, we included CDAI measurement within the primary outcome even though it has been previously criticised in this setting. The 36-item Short Form quality-of-life instrument underwent internal text changes at the time of trial start-up. The reporting of these results was deemed not to be compliant with 36-item Short Form licensing terms and these results are therefore not presented.

A definitive study of postoperative prevention of Crohn's disease has proved difficult to undertake. The PREVENT study^24^ was terminated early because of small numbers of patients reaching the primary outcome. The PREVENT study^24^ had selective inclusion criteria, and no difference was reported in clinical relapse between those on infliximab and those on placebo at week 76, although an endoscopic effect was noted. A smaller study^25^ that compared early azathioprine initiation with azathioprine driven by endoscopic findings at week 26 was stopped after 6 years because of slow recruitment, with no meaningful conclusions. Although our study was underpowered to detect the reported treatment effect, and many patients were under-dosed with mercaptopurine, our data nonetheless provide some evidence of efficacy of mercaptopurine in the context of postoperative prevention. Indeed, a meta-analysis of these data with the two other randomised placebo-controlled trials of thiopurines in the postoperative setting^16^, ^19^ shows a significant reduction in postoperative clinical relapse at 12 months (relative risk 0·57, 95% CI 0·38–0·85; appendix p 11). Taken with the other recent data, our study helps to make progress towards a treatment algorithm for all patients after surgery for Crohn's disease, with smoking habit the key determinant affecting management.

Several areas now require further clinical studies, including putative mechanisms for the effect of smoking on Crohn's disease,^26^ and smoking intervention studies. The efficacy and safety of mercaptopurine compared with anti-tumour-necrosis-factor (TNF) as postoperative preventive treatment is a key issue to investigate. At present, anti-TNF treatment is reserved for patients who are intolerant or unresponsive to thiopurine, but the safety, efficacy, and cost of these drugs is under continuous reassessment. Endoscopic findings and faecal calprotectin remain important components of disease assessment, but the exact parameters that best define postoperative recurrent disease remain to be elucidated.
