# Supplementary material for: Mercaptopurine versus placebo to prevent recurrence of Crohn's disease after surgical resection (TOPPIC): a multicentre, double-blind, randomised controlled trial
Source: Lancet Gastroenterol Hepatol. 2016 Aug 30;1(4):273–82. doi: 10.1016/S2468-1253(16)30078-4 (PMC6358144; doi:10.1016/S2468-1253(16)30078-4)

THE LANCET
Gastroenterology & Hepatology

Supplementary appendix

This appendix formed part of the original submission and has been peer reviewed. We post it as supplied by the authors.

Supplement to: Mowat C, Arnott I, Cahill A, et al, for the TOPPIC Study Group. Mercaptopurine versus placebo to prevent recurrence of Crohn's disease after surgical resection (TOPPIC): a multicentre, double-blind, randomised controlled trial. *Lancet Gastroenterol Hepatol* 2016; published online Aug 30. http://dx.doi.org/10.1016/S2468-1253(16)30078-4.

TOPPIC Web appendix

Contents

TOPPIC Study Group members 2

Supplementary table 1: Inclusion and Exclusion Criteria for the TOPPIC Trial 4

Supplementary table 2: Dose reduction algorithm 5

Supplementary table 3: Overview of Trial Procedures 6

Supplementary table 4: Recruitment to TOPPIC per centre 7

Supplementary table 5: Data and sample completion rates across TOPPIC trial outcomes 8

Supplementary table 6: Sensitivity and specificity (and 95% CIs) of faecal calprotectin for prediction of post-operative endoscopic recurrence of Crohn's disease (Rutgeerts' ≥ i2) 9

Supplementary table 7: Adverse events stratified by category, severity and treatment group 10

Supplementary figure 1: Meta-analysis of published randomised controlled trial data comparing thiopurines to placebo for prevention of post-operative recurrence within 12 months in Crohn's disease 11

Supplementary figure 2: Faecal calprotectin concentrations measured at weeks 49 and 157 plotted against corresponding Rutgeerts' score for endoscopic recurrence of Crohn's disease 12

Supplementary figure 3: Thioguanine nucleotide (TGN) blood concentrations in patients receiving MP plotted by study week, and grouped according to therapeutic range (<235 – 450, pmol/8x108 RBC), n=128 13

Supplementary figure 4: Endoscopic recurrence of Crohn's disease as measured by Rutgeerts' score at Week 49 post-randomisation in patients on mercaptopurine (MP) vs placebo 14

Supplementary figure 5: Endoscopic recurrence of Crohn's disease as measured by Rutgeerts' score at Week 157 post-randomisation in patients on mercaptopurine (MP) vs placebo comparing MP vs placebo 15

TOPPIC Study Group members

Site teams

Aberdeen Royal Infirmary, Abderdeen
Malcolm Smith[4], Aileen J McKinley[29], John M Thomson[4], Ashley Mowat[4]

Barts and the London, London
James O Lindsay[26], Louise Langmead[26]

Bristol Royal Infirmary, Bristol
Tom Creed[13]

Darlington Memorial Hospital, Darlington and University Hospital of North Durham, Durham
Anjan Dhar[10]

Derriford Hospital, Plymouth
Stephen J Lewis[18]

Glasgow Royal Infirmary, Glasgow
Aiden Cahill[3], John Morris[3], Daniel R Gaya[3], Jack Winter[3], Graham D Naismith[31]

Hull Royal Infirmary, Hull
Shaji Sebastian[19]

John Radcliffe Hospital, Oxford
Simon Travis[8]

Leeds General Infirmary, Leeds
John Hamlin[9]

Manchester Royal Infirmary, Manchester
Simon Campbell[17], Scott Levison[17]

Ninewells, Dundee
Craig Mowat[1], John A Todd[1]

North Staffs, Stoke-on-Trent (now Royal Stoke University Hospital)
Sandip Sen[20]

Queen Elizabeth Hospital, Birmingham
Jason Goh[25]

Queen's Medical Centre, Nottingham
Chris Hawkey[22]

Raigmore, Inverness
Lindsay Potts[28]

Rotherham General Hospital, Rotherham
Mohamed Yousif[15], Pierre Willemse[15]

Royal Devon and Exeter Hospital, Exeter
Tariq Ahmad[5,6]

Royal Free, London
Charles Murray[23]

Royal Liverpool University Hospital, Liverpool
Sreedhar Subramanian[7]

Salford Royal Infirmary, Salford
Simon Lal[21]

Singleton Hospital, Swansea
Linzi Thomas[16]

Southampton General Hospital, Southampton
Fraser Cummings[24]

St Marks, London
Naila Arebi[27]

Torbay Hospital, Torquay
Cathryn Edwards[12]

UCLH, London
Stuart Bloom[14]

University Hospital Coventry, Coventry
Chuka Nwokolo[11]

Western General Hospital, Edinburgh
Ian Arnott[2], Mhairi Collie[30], Malcolm G Dunlop[30], Nicholas A Kennedy[2,6], Jack Satsangi[2], Charlie Lees[2], David Bartolo[30,34]

Trial steering committee
John Mansfield[35], Chris SJ Probert[36], Stuart Ralston[37], Ruth Slater

Data monitoring committee
Stephen JW Evans[38], Helen Gillett[39], Huw Roddie[40]

ECTU
Holly Ennis[32], Gail Campbell[32], Audrey Duncan[32], Audrey Duncan[32], Debra Kerr[32], Ashma Krishan[32], Garry Milne[32], Lynsey Milne[32], Gail Scott[32], Samantha Thomas[32], Allan Walker[32], Helen Watters[32]

ACCORD
Marise Bucukoglu[41], Anne Langston[41]

Statistical team
Catriona Keerie[32], Steff Lewis[32], Robin J Prescott[33]

Laboratory teams

Cedars-Sinai F. Widjaja Foundation Inflammatory Bowel and Immunobiology Research Institute, Cedars-Sinai Medical Centre, Los Angeles
Michelle (Xiaoxiao) Li[44], Dermot McGovern[44]

Dept of Clinical Chemistry, Western General Hospital, Edinburgh
Ann Jarvie[43], Julie Nichols[43], Susan A Walker[43]

Dept of Pathology, Western General Hospital, Edinburgh
David Worrall[46]

Purine Research Laboratory, St Thomas' Hospital, London
Tony Marinaki[45]

Wellcome Trust Clinical Research Facility, University of Edinburgh, Edinburgh
Mark Blandford[42], Lee Murphy[42]

[1]Gastrointestinal Unit, Ninewells Hospital, Dundee, UK. [2]Gastrointestinal Unit, Western General Hospital, Edinburgh, UK. [3]Gastrointestinal Unit, Glasgow Royal Infirmary, Glasgow, UK. [4]Gastrointestinal Unit, Aberdeen Royal Infirmary, Abderdeen, UK. [5]Dept of Gastroenterology, Royal Devon and Exeter Hospital NHS Foundation Trust, Exeter, UK. [6]IBD Pharmacogenetics Unit, University of Exeter, Exeter, UK. [7]Dept of Gastroenterology, Royal Liverpool University Hospital, Liverpool, UK. [8]Translational Gastroenterology Unit, Nuffield Department of Experimental Medicine, University of Oxford, Oxford, UK. [9]Dept of Gastroenterology, Leeds General Infirmary, Leeds Teaching Hospitals NHS Trust, Leeds, UK. [10]Dept of Gastroenterology, Darlington Memorial Hospital, Darlington, UK. [11]Dept of Gastroenterology, University Hospital Coventry and Warwickshire NHS Trust, Coventry, UK. [12]Dept of Gastroenterology, Torbay Hospital, South Devon Healthcare NHS Foundation Trust, Torbay, Devon, UK. [13]Dept of Gastroenterology, Bristol Royal Infirmary, University Hospitals Bristol NHS Foundation Trust, Bristol, UK. [14]Dept of Gastroenterology, University College London Hospitals NHS Foundation Trust, London, UK. [15]Dept of Gastroenterology, Rotherham NHS Foundation Trust Hospital, Rotherham, UK. [16]Dept of Gastroenterology, Singleton Hospital, Abertawe Bro Morgannwg University Health Board, Swansea, UK. [17]Dept of Gastroenterology, Manchester Royal Infirmary, Central Manchester University Hospitals NHS Foundation Trust, Manchester, UK. [18]Dept of Gastroenterology, Derriford Hospital, Plymouth Hospitals NHS Trust, Plymouth, UK. [19]Dept of Gastroenterology, Hull Royal Infirmary, Hull and East Yorkshire Hospitals NHS Trust, Hull, UK. [20]Dept of Gastroenterology, Royal Stoke University Hospital, University Hospitals of North Midlands NHS Trust, Stoke-on-Trent, UK. [21]Dept of Gastroenterology, Salford Royal NHS Foundation Trust Hospital, Salford, UK. [22]Dept of Gastroenterology, Nottingham University Hospitals NHS Trust, Nottingham, UK. [23]Dept of Gastroenterology, Royal Free London NHS Foundation Trust Hospital, London, UK. [24]Dept of Gastroenterology, Southampton General Hospital, University Hospital Southampton NHS Foundation Trust, Southampton, UK. [25]Dept of Gastroenterology, Queen Elizabeth Hospital, University Hospitals Birmingham NHS Foundation Trust, Birmingham, UK. [26]Dept of Gastroenterology, Barts Health NHS Trust, Barts and the London School of Medicine, London, UK. [27]Inflammatory Bowel Disease Unit, St Mark’s Hospital, North West London Hospitals NHS Trust, London, UK. [28]Gastrointestinal Unit, Raigmore Hospital, Inverness, UK. [29]Dept of Surgery, Aberdeen Royal Infirmary, Abderdeen, UK. [30]Colorectal Surgery, Western General Hospital, Edinburgh, UK. [31]Gastrointestinal Unit, Princess Alexandra Hospital, Paisley, UK. [32]Edinburgh Clinical Trials Unit, Usher Institute, University of Edinburgh, Edinburgh, UK. [33]Usher Institute, University of Edinburgh, Edinburgh, UK. [34]Dept of Colorectal Surgery, Fiona Stanley Hospital, Murdoch, WA, Australia. [35]Dept of Gastroenterology, Victoria Royal Infirmary, Newcastle, UK. [36]Gastroenterology, Cellular and Molecular Physiology, University of Liverpool, Liverpool, UK. [37]Rheumatology and Bone Disease, Institute of Genetics and Molecular Medicine, University of Edinburgh, Edinburgh, UK. [38]Department of Medical Statistics, London School of Hygiene and Tropical Medicine, London, UK. [39]Gastrointestinal Unit, Royal Hospital for Sick Children, Edinburgh, UK. [40]Dept of Haematology, Western General Hospital, Edinburgh, UK. [41]ACCORD, University of Edinburgh, Edinburgh, UK. [42]Wellcome Trust Clinical Research Facility, University of Edinburgh, Edinburgh, UK. [43]Dept of Clinical Chemistry, Western General Hospital, Edinburgh, UK. [44]Cedars-Sinai F. Widjaja Foundation Inflammatory Bowel and Immunobiology Research Institute, Cedars-Sinai Medical Centre, Los Angeles, California, USA. [45]Purine Research Laboratory, St Thomas' Hospital, London, UK. [46]Dept of Pathology, Western General Hospital, Edinburgh, UK.

Supplementary table 1: Inclusion and Exclusion Criteria for the TOPPIC Trial

**Inclusion Criteria**
A patient will be considered eligible for inclusion in the study if all of the following criteria apply:<br><br>1. At least 16 years of age in Scotland and 18 years of age in England and Wales.<br>2. Established diagnosis of Crohn's disease confirmed at recent resection.<br>3. Ileocolonic or small bowel resection within 3 months before screening.<br>4. No more than 100 cm of fixed small bowel resected in total. Previous ileocolonic resection is acceptable.<br>5. Able to start oral nutrition within the first 2 postoperative weeks.<br>6. Normal or heterozygous TPMT (activity present or reduced consistent with carrier status).<br>7. Able to provide written informed consent prior to screening and to comply with the requirements of the study protocol.<br>8. Off antibiotics 2 weeks prior to randomisation.
**Exclusion Criteria**
A patient will not be eligible for inclusion in the study if any of the following criteria apply:<br><br>1. Pregnancy at baseline or breast feeding.<br>2. A known hypersensitivity or intolerance to 6MP<br>3. Pancreatitis associated with azathioprine.<br>4. Receiving an experimental treatment for Crohn's disease in the 4 weeks prior to study entry.<br>5. Known to require further surgery at study entry i.e. for the removal of an abscess developing from the primary surgery.<br>6. Strictureplasty procedure alone<br>7. (Please note that strictureplasty and resection procedure together will not be considered an exclusion.)<br>8. Presence of stoma.<br>9. Significant haematological, renal or hepatic dysfunction or clinically important lung disease (i.e. liver function tests (except GGT) >x2 upper limit of normal, Haemoglobin ≤10, total white blood cell count <3.5, Neutrophils <1.5, Platelets $<100x10^9/l$).<br>10. Systemic infection including hepatitis B, hepatitis C, HIV and active TB.<br>11. A diagnosis of indeterminate colitis or ulcerative colitis.<br>12. A history of illicit drug or alcohol abuse in the 1 year prior to study entry.<br>13. Active or untreated malignancy (excluding basal cell carcinoma and insitu tumours). (Patients who have had successful treatment for malignancy and have been in remission for more than 5 years may be considered for inclusion only after detailed discussion with, and written approval, from the patient's medical oncologist.)<br>14. Presence of a medical or psychiatric condition, disease or laboratory abnormality that in the opinion of the PI may place the subject at unacceptable risk during the study.<br>15. Homozygous deficient for TPMT (absent activity)<br>16. Evidence of untreated post-operative infection e.g. clostridium difficile, urinary tract infection or chest infection. If these have been appropriately treated in the opinion of the PI, and inclusion criteria 8 is met, this will not be considered an exclusion.<br>17. Taking any medication for Crohn's disease.

Supplementary table 2: Dose reduction algorithm

**Option 1**	No action required as not deemed to be a safety issue (Some abnormal results will be at the discretion of the Investigators as to whether they require any action.)
**Option 2**	Hb<10.0: E-mail local nurse to telephone patient and arrange for review and assessment by Investigator as would be done in routine clinical practice. Anaemia could be caused by a number of reasons so this should not affect the study blind.
**Option 3**	Total White Cell Count 2.5-3.5, **or** Neutrophil count between 1.0 and 2.0 **or** any LFT (except GGT) between >2 and 5 times the upper limit of normal **or** Platelet count between 50-100x10^6/l:<br>Email local nurse who will telephone patient to reduce dose. Ensure safety bloods are done within 1 week at local site. This should not affect the study blind.
**Option 4**	Total White Cell Count <2.5 or Neutrophil count <1.0 or Any LFT >x5 upper limit of normal or platelet count <50x106/l: Email and telephone local nurse who will telephone patient to discontinue the study drug, arrange for immediate patient review by Investigator and repeat bloods, at weekly intervals, until resolved. At the duty clinicians' discretion drug could be recommenced or the dose reduced [see Para 7.7 of Protocol Dose Reduction Schedule (Table 2)], if out of range values improve above the lower limits designated. Patient will continue as per protocol. Otherwise study drug would be stopped and patient should continue with scheduled visits but not take any more IMP. (The breaking of the study blind will only be performed where knowledge of the treatment is absolutely necessary for further management of the patient).

Supplementary table 3: Overview of Trial Procedures

**Visit number**	**-1**	**1**	**2**	**3**	**4**	**5**	**6**	**7**	**8**	**9**	**10**	**11**	**12**
**Study Week No**	**-4**	**-1**	**0**	**6**	**13**	**31**	**49**	**67**	**85**	**103**	**121**	**139**	**157**
**Visit purpose**	**Inpatient pre-assessment**	**Safety Visit**	**Randomisation and drug prescription**	**Interim safety assessment**	**Study visit**	**Study visit**	**Study visit**	**Study visit**	**Study visit**	**Study visit**	**Study visit**	**Study visit**	**Final study visit**
Inc/Exc Criteria	x												
Consent Form	x		x										
Patient Information Leaflet	x												
TPMT	x												
Stool Sample	x												
Pregnancy Test			x										
Clinic Visit			x	x	x	x	x	x	x	x	x	x	x
Medical History			x										
CDAI (Diary to be completed by patient 7 days prior to this visit)			x	x	x	x	x	x	x	x	x	x	x
Weight			x	x	x	x	x	x	x	x	x	x	x
Height			x										
Study Drug			x	x	x	x	x	x	x	x	x	x	
Physical exam inc vital signs			x	x	x	x	x	x	x	x	x	x	x
AEs		x	x	x	x	x	x	x	x	x	x	x	x
Blood Safety Monitoring*		x		x	x	x	x	x	x	x	x	x	x
6MP Metabolite Levels			x		x		x			x			x
Faecal Calprotectin			x		x		x			x			x
Colonoscopy & Biopsy							x						x
IBDQ, SF-36 & EQ-5D			x		x		x			x			x
Prohibited Meds				x	x	x	x	x	x	x	x	x	x
Genetic Studies			X										
Serological Studies			X										

Supplementary table 4: Recruitment to TOPPIC per centre

Centre	Principal Investigator	Number of patients	% of patients
**Western General Hospital**, Edinburgh	Dr Ian Arnott	78	32.5
**Ninewells**, Dundee	Dr Craig Mowat	21	8.8
**Stobhill Hospital**, Glasgow	Dr Aidan Cahill	19	7.9
**Aberdeen Royal Infirmary**, Abderdeen	Dr Malcolm Smith	17	7.1
**Royal Devon and Exeter Hospital**, Exeter	Dr Tariq Ahmad	11	4.6
**John Radcliffe Hospital**, Oxford	Dr Simon Travis	9	3.8
**Glasgow Royal Infirmary**, Glasgow	Dr John Morris	8	3.3
**Royal Liverpool University Hospital**, Liverpool	Sreedhar Subramanian	8	3.3
**Leeds General Infirmary**, Leeds	Dr John Hamlin	8	3.3
**University Hospital Coventry**, Coventry	Professor Chuka Nwokolo	6	2.5
**Torbay Hospital**, Torquay	Dr Cathryn Edwards	6	2.5
**University Hospital of North Durham**, Durham	Dr Anjan Dhar	6	2.5
**Bristol Royal Infirmary**, Bristol	Dr Tom Creed	5	2.1
**UCLH**, London	Dr Stuart Bloom	5	2.1
**Singleton Hospital**, Swansea	Dr Linzi Thomas	4	1.7
**Rotherham General Hospital**, Rotherham	Dr Mohamed Yousif	4	1.7
**Manchester Royal Infirmary**, Manchester	Dr Simon Campbell	3	1.2
**Derriford Hospital**, Plymouth	Dr Stephen Lewis	3	1.2
**Hull Royal Infirmary**, Hull	Dr Shaji Sebastian	3	1.2
**Salford Royal Infirmary**, Salford	Dr Simon Lal	2	0.8
**Royal Free**, London	Dr Charles Murray	2	0.8
**Southampton General Hospital**, Southampton	Dr Fraser Cummings	2	0.8
**Queen's Medical Centre**, Nottingham	Professor Chris Hawkey	2	0.8
**Queen Elizabeth Hospital**, Birmingham	Dr Jason Goh	2	0.8
**Darlington Memorial Hospital**, Darlington	Dr Anjan Dhar	2	0.8
**Barts and the London**, London	Dr James Lindsay	1	0.4
**North Staffs**, Stoke-on-Trent (now Royal Stoke University Hospital)	Dr Sandip Sen	1	0.4
**St Marks**, London	Dr Naila Arebi	1	0.4
**Raigmore**, Inverness	Dr Lindsay Potts	1	0.4

Supplementary table 5: Data and sample completion rates across TOPPIC trial outcomes

Parameter	Timepoint post-randomisation	6MP (n=128)	Placebo (n=112)	Overall (n=240)
**Clinical data**	**Week 0**	128 (100)	112 (100)	240 (100)
**Clinical data**	**Week 6**	123 (96.1)	108 (96.4)	231 (96.3)
**Clinical data**	**Week 13**	117 (91.4)	107 (95.5)	224 (93.3)
**Clinical data**	**Week 31**	114 (89.1)	99 (88.4)	213 (88.8)
**Clinical data**	**Week 49**	111 (86.7)	97 (86.6)	208 (86.7)
**Clinical data**	**Week 67**	105 (82.0)	87 (77.7)	192 (80.0)
**Clinical data**	**Week 85**	100 (78.1)	83 (74.1)	183 (76.3)
**Clinical data**	**Week 103**	98 (76.6)	78 (69.6)	176 (73.3)
**Clinical data**	**Week 121**	94 (73.4)	74 (66.1)	168 (70.0)
**Clinical data**	**Week 139**	89 (69.5)	71 (63.4)	160 (66.7)
**Clinical data**	**Week 147**	89 (69.5)	71 (63.4)	161 (67.1)
**Colonoscopy**	**Week 49**	95 (74)	77 (69)	172 (72)
**Colonoscopy**	**Week 157**	69 (54)	59 (53)	128 (53)
**Calprotectin**	**Week 0**	108	96	204
**Calprotectin**	**Week 13**	94	87	181
**Calprotectin**	**Week 49**	87	67	154
**Calprotectin**	**Week 103**	69	55	124
**Calprotectin**	**Week 157**	65	52	117
**6-TGN**	**Week 0**	124	110	234
**6-TGN**	**Week 13**	114	97	211
**6-TGN**	**Week 49**	102	86	188
**6-TGN**	**Week 103**	82	65	147
**6-TGN**	**Week 157**	64	57	121
**IBDQ**	**Week 0**	128	111	239
**IBDQ**	**Week 13**	117	106	223
**IBDQ**	**Week 49**	109	94	203
**IBDQ**	**Week 103**	97	75	172
**IBDQ**	**Week 157**	86	69	155

Supplementary table 6: Sensitivity and specificity (and 95% CIs) of faecal calprotectin for prediction of post-operative endoscopic recurrence of Crohn's disease (Rutgeerts' ≥ i2)

**Endoscopic Parameter**	FC cut-off (μg/g)	Sensitivity	Specificity	PPV	NPV
**Recurrence (≥i2)**	50	84.4% (77.0%, 91.9%)	44.4% (35.6%, 53.1%)	52.4% (44.3%, 60.5%)	79.7% (70.2%, 89.2%)
	100 (optimum*)	72.2% (63.0%, 81.5%)	62.1% (53.6%, 70.6%)	58.0% (48.9%, 67.2%)	75.5% (67.1%, 83.8%)
**Remission (i0)**	50	51.8% (38.7%, 64.9%)	74.7% (67.9%, 81.5%)	42.0% (30.4%, 53.7%)	81.4% (75.0%, 87.7%)
	100	67.9% (55.6%, 80.1%)	59.5% (51.8%, 67.2%)	37.3% (27.9%, 46.6%)	83.9% (77.1%, 90.7%)
	60 (optimum*)	55.4% (42.3%, 68.4%)	70.9% (63.8%, 78.0%)	40.3% (29.3%, 51.2%)	81.8% (75.3%, 88.2%)

These data derived from combined Visit 6 (week 49) and Visit 12 (week 157) data (n = 214)

* Optimum cut-off point calculated by maximising Youden's J statistic.

Supplementary table 7: Adverse events stratified by category, severity and treatment group

	Mercaptopurine				Placebo			
**Category**	**Mild**	**Moderate**	**Severe**	**Total**	**Mild**	**Moderate**	**Severe**	**Total**
**Cancers**	0	3	0	**3**	0	0	1	**1**
**Deranged LFTs**	0	4	0	**4**	0	5	0	**5**
**GI Symptoms - Abdominal pain**	41	64	26	**132**	32	85	24	**141**
**GI Symptoms - Constipation/diarrhoea**	23	24	7	**54**	17	31	8	**56**
**GI Symptoms - Nausea/vomiting**	25	42	11	**78**	18	21	2	**41**
**GI Symptoms - Other**	23	26	4	**53**	22	18	0	**40**
**Headache**	39	22	0	**61**	17	18	3	**38**
**Infections**	85	80	6	**171**	75	102	7	**184**
**Joint pain/arthralgia**	28	40	4	**72**	21	40	4	**65**
**Pain**	11	15	4	**30**	4	12	3	**19**
**Pancreatitis**	0	1	0	**1**	0	0	1	**1**
**Rash**	21	12	2	**35**	15	2	0	**17**
**Worsening Crohn's**	6	29	6	**41**	5	25	7	**37**
**Other**	97	106	9	**212**	70	72	11	**153**

Numbers indicate total number of events. There was one case of abdominal pain in the mercaptopurine group with missing severity, and one mild event with a missing category.

Supplementary figure 1: Meta-analysis of published randomised controlled trial data comparing thiopurines to placebo for prevention of post-operative recurrence within 12 months in Crohn's disease

FE = Fixed effects

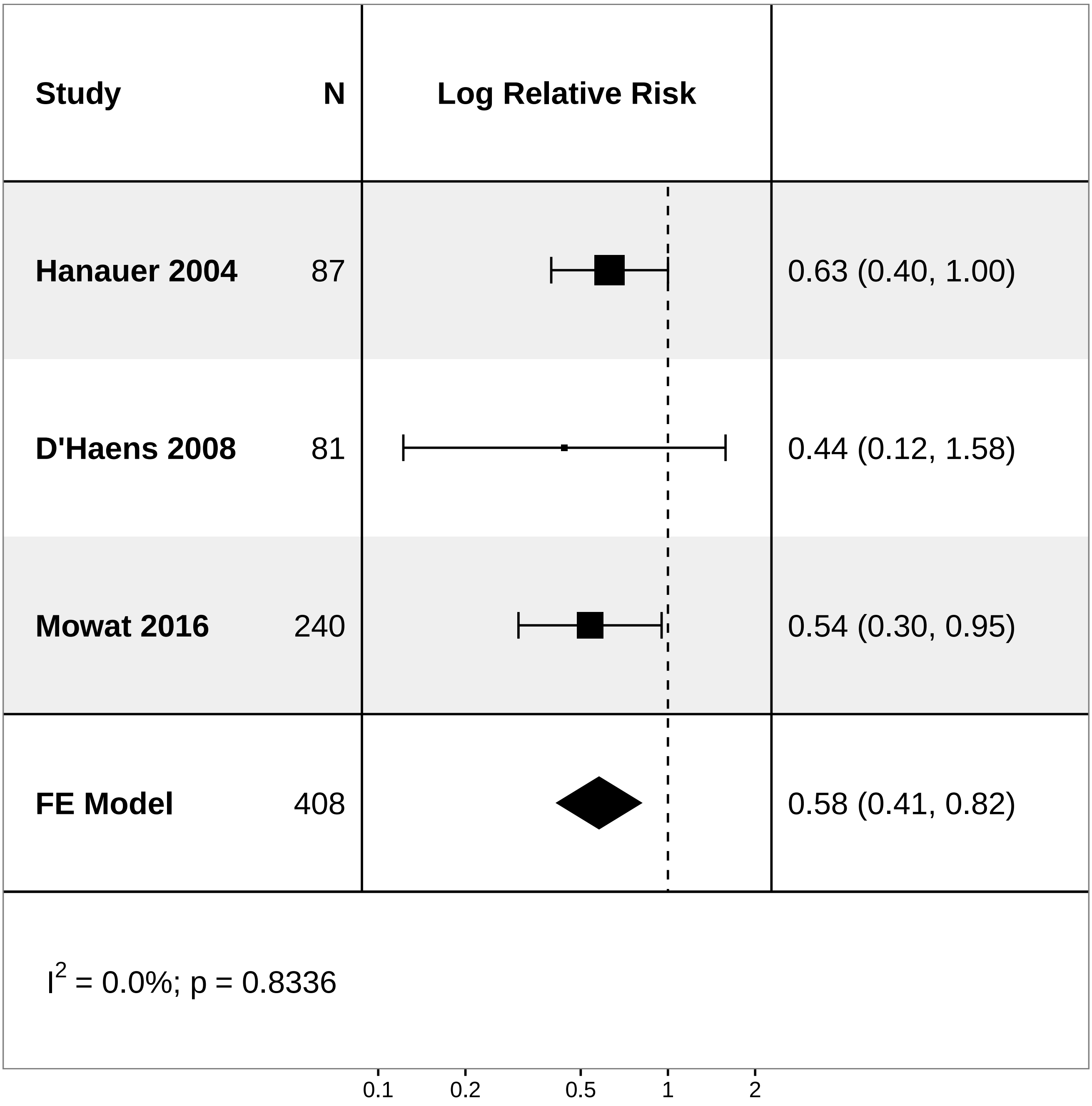

Supplementary figure 2: Faecal calprotectin concentrations measured at weeks 49 and 157 plotted against corresponding Rutgeerts' score for endoscopic recurrence of Crohn's disease

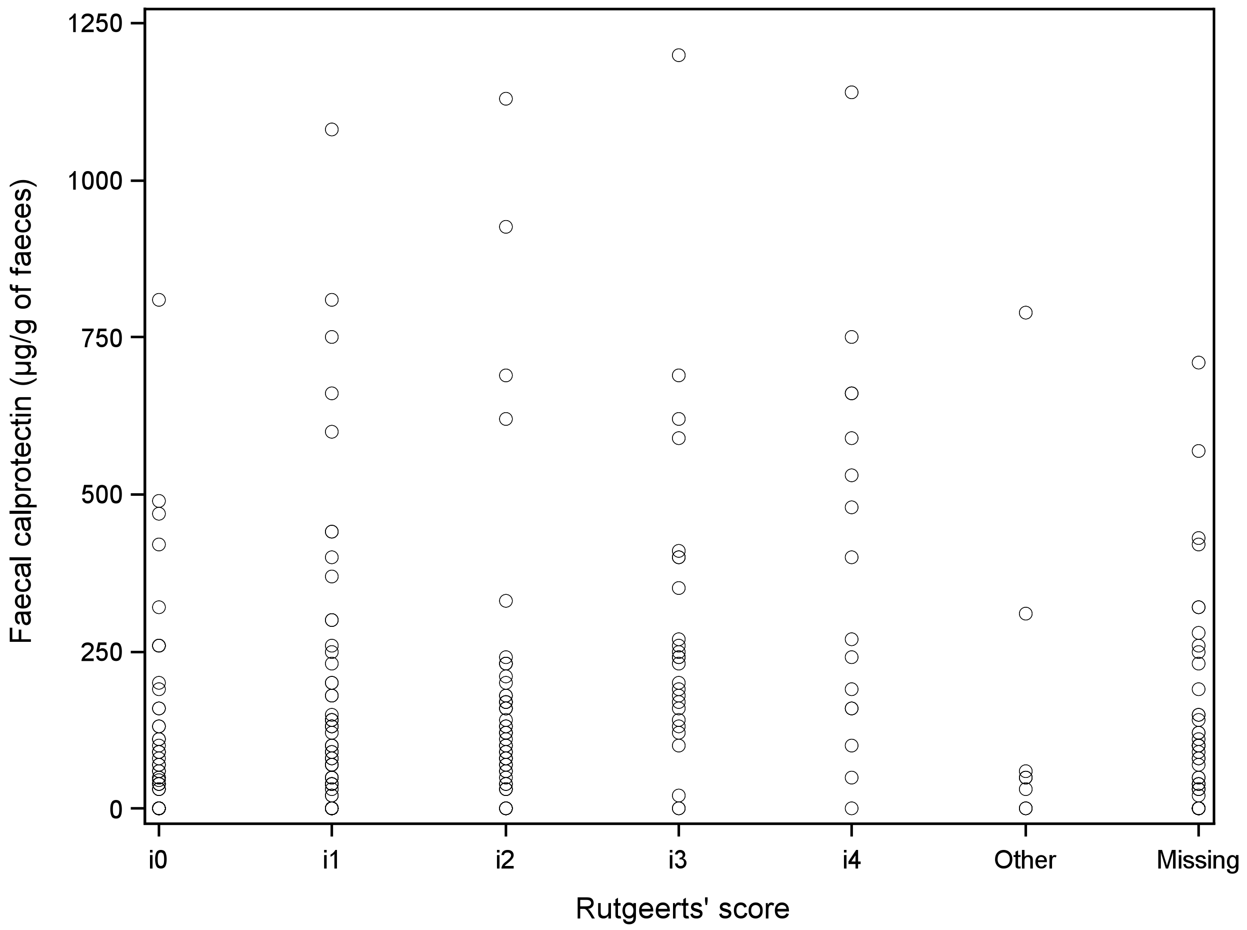

Supplementary figure 3: Thioguanine nucleotide (TGN) blood concentrations in patients receiving MP plotted by study week, and grouped according to therapeutic range (<235 – 450, pmol/8x108 RBC), n=128

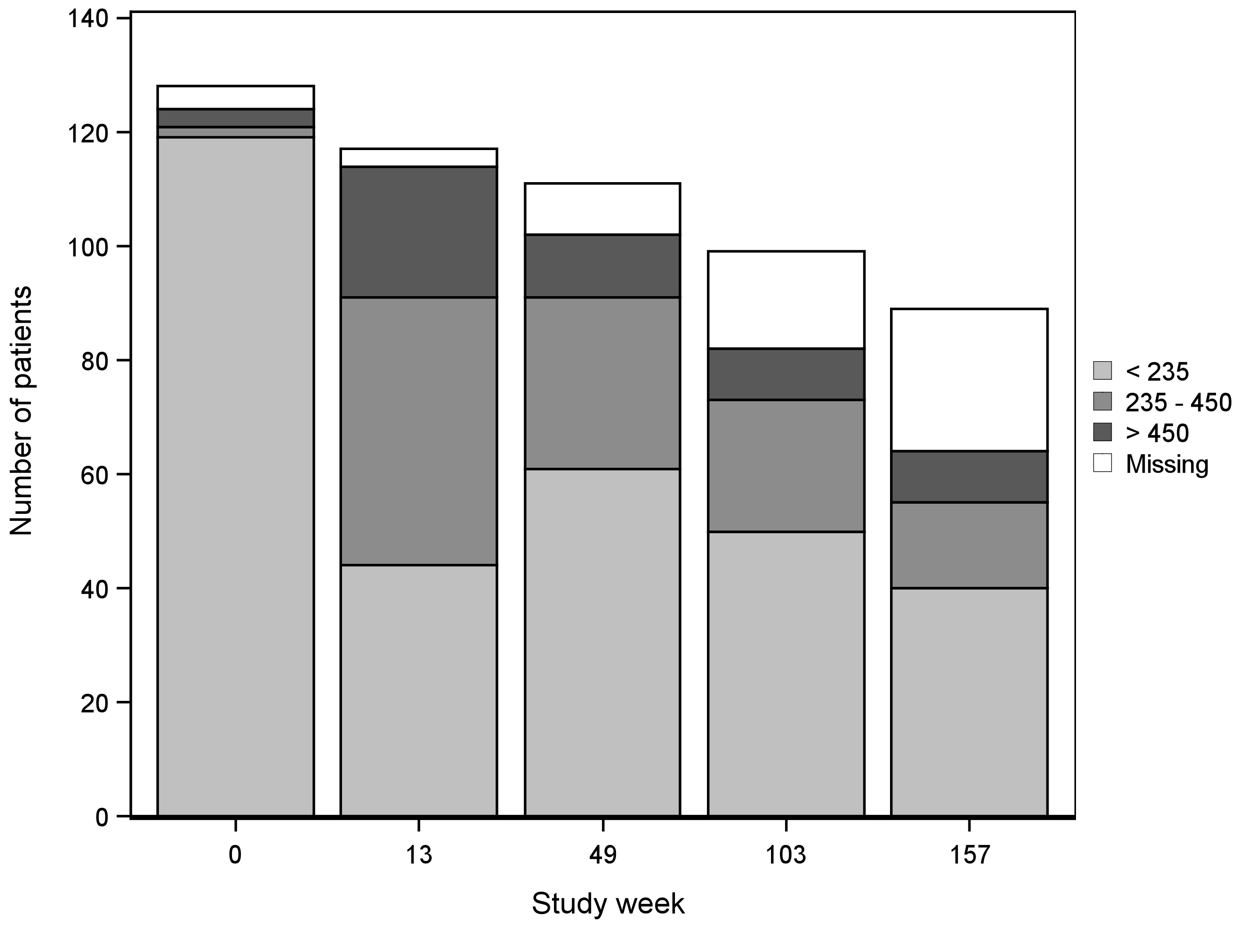

Supplementary figure 4: Endoscopic recurrence of Crohn's disease as measured by Rutgeerts' score at Week 49 post-randomisation in patients on mercaptopurine (MP) vs placebo

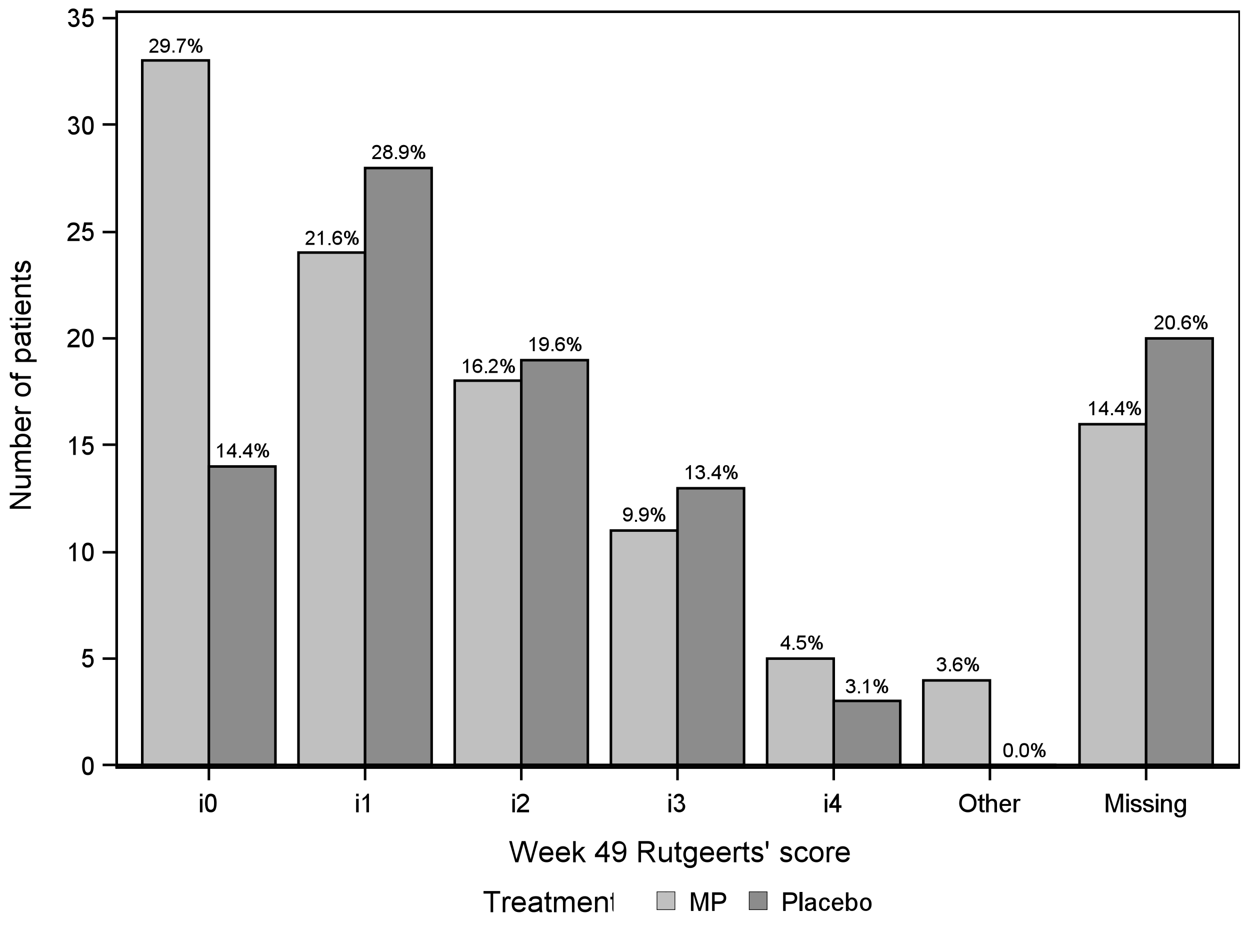

Supplementary figure 5: Endoscopic recurrence of Crohn's disease as measured by Rutgeerts' score at Week 157 post-randomisation in patients on mercaptopurine (MP) vs placebo comparing MP vs placebo

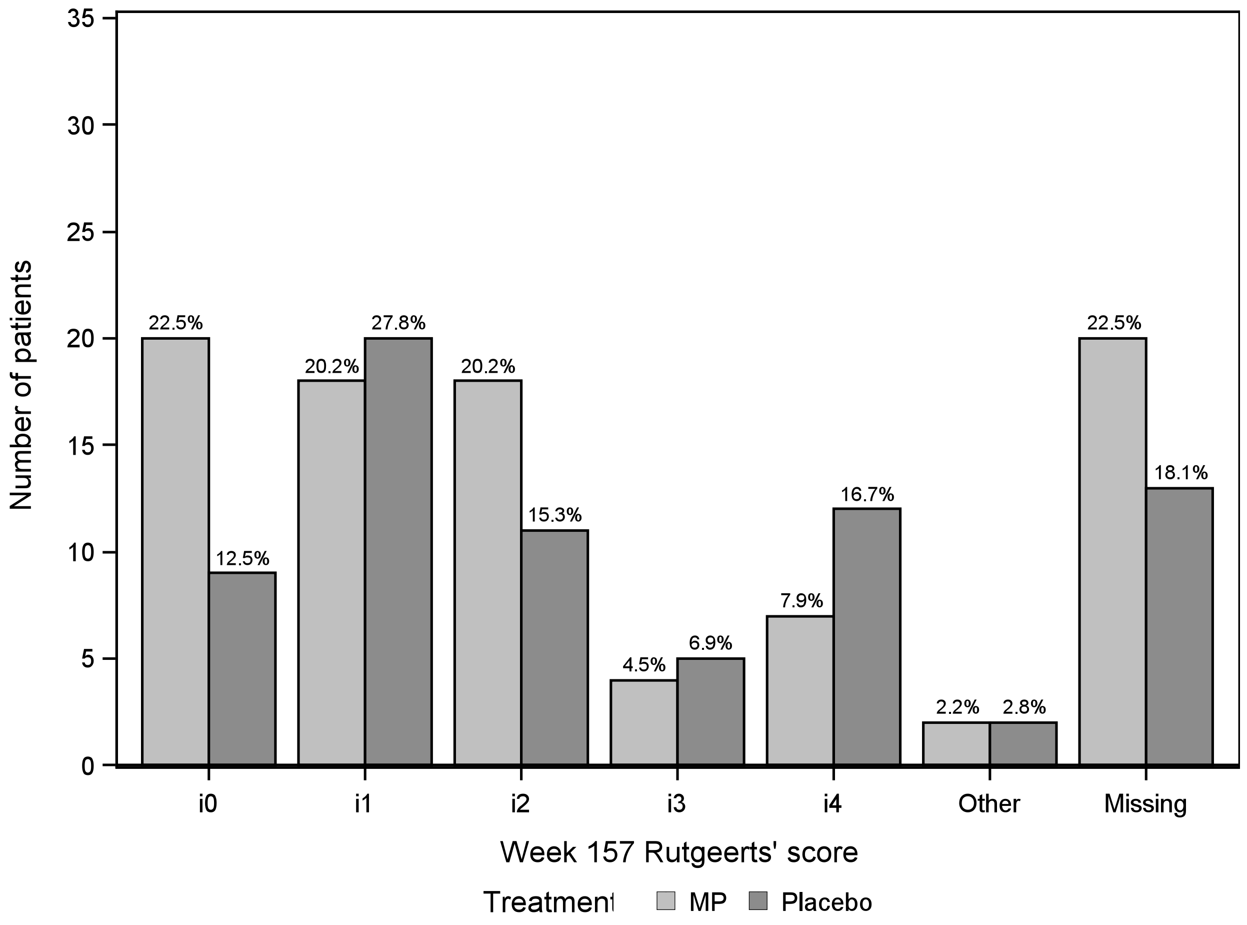

Supplement: Supplementary appendix [file mmc1.pdf]
